# miR-10-3p, but not miR-1006-3p, regulates *mei-P26* expression in the *Drosophila* testis

**DOI:** 10.17912/micropub.biology.002187

**Published:** 2026-08-31

**Authors:** Shallinie Thangadurai, Elena Pak, Isabella Hammock, Alexander Murashov

**Affiliations:** 1 Comparative Biomedical Sciences, Louisiana State University, Baton Rouge, LA, United States; 2 Saint Joseph's Academy

## Abstract

MicroRNAs (miRNAs) are key post-transcriptional regulators of gene expression, yet predicted miRNA target interactions often require experimental validation. TargetScan Fly predicts that both miR-10-3p and miR-1006-3p target the *Drosophila* RNA-binding protein Mei-P26, a critical regulator of germline development and miRNA biogenesis. Here, we experimentally demonstrate that overexpression of miR-10-3p is sufficient to significantly suppress *mei-P26* mRNA level in the testis. Consistently, inhibition of miR-10 using sponge increased mei-P26 positive spermatocytes in testis. Together, these findings suggest that miR-10-3p, but not miR-1006-3p, functionally regulates *mei-P26* in *Drosophila* testis, highlighting selective engagement of canonical miRNA pathways in *mei-P26* regulation.

**Figure 1. Expression of mei-P26 in Drosophila testis is regulated by miR-10-3p, not miR-1006-3p although they share the same seed sequence:** (A) Mei-P26 and miRNA target enrichment analysis of proteomic data using MIENTURNET showing miR-10-3p and miR-1006-3p as potential miRNAs that target *mei-P26* . (B) Predicted conserved miRNA regulators of *mei-P26* using TargetScanFly 7.2. (C) Testis tissue specific expression level of top predicted miRNAs in reads per million mapped miRNA reads (RPMM). (D) Mei-P26 3’UTR binding site targeted by miR-10-3p and miR-1006-3p seed sequence. (E-G) RT-qPCR showing relative fold change of *mei-P26* , miR-10-3p and miR-1006-3p in *Drosophila* testis with germline overexpression of *mei-P26* , miR-10 and miR-1006. Statistical significance was assessed using unpaired two-tailed Student’s *t* -test. * = *p* < 0.05; ** = *p* < 0.005; *** = *p* < 0.0005; **** = *p* < 0.0001 using GraphPad Prism 10. (H-J) Immunostaining of *Drosophila* testis with DAPI (in blue) marking DNA, Mei-P26 (in red) marking spermatocytes, Caspase 3 marking waste bags and alpha-spectrin marking fusomes (in green). ‘*’ denotes testicular hub. Image frame: 200 μm.

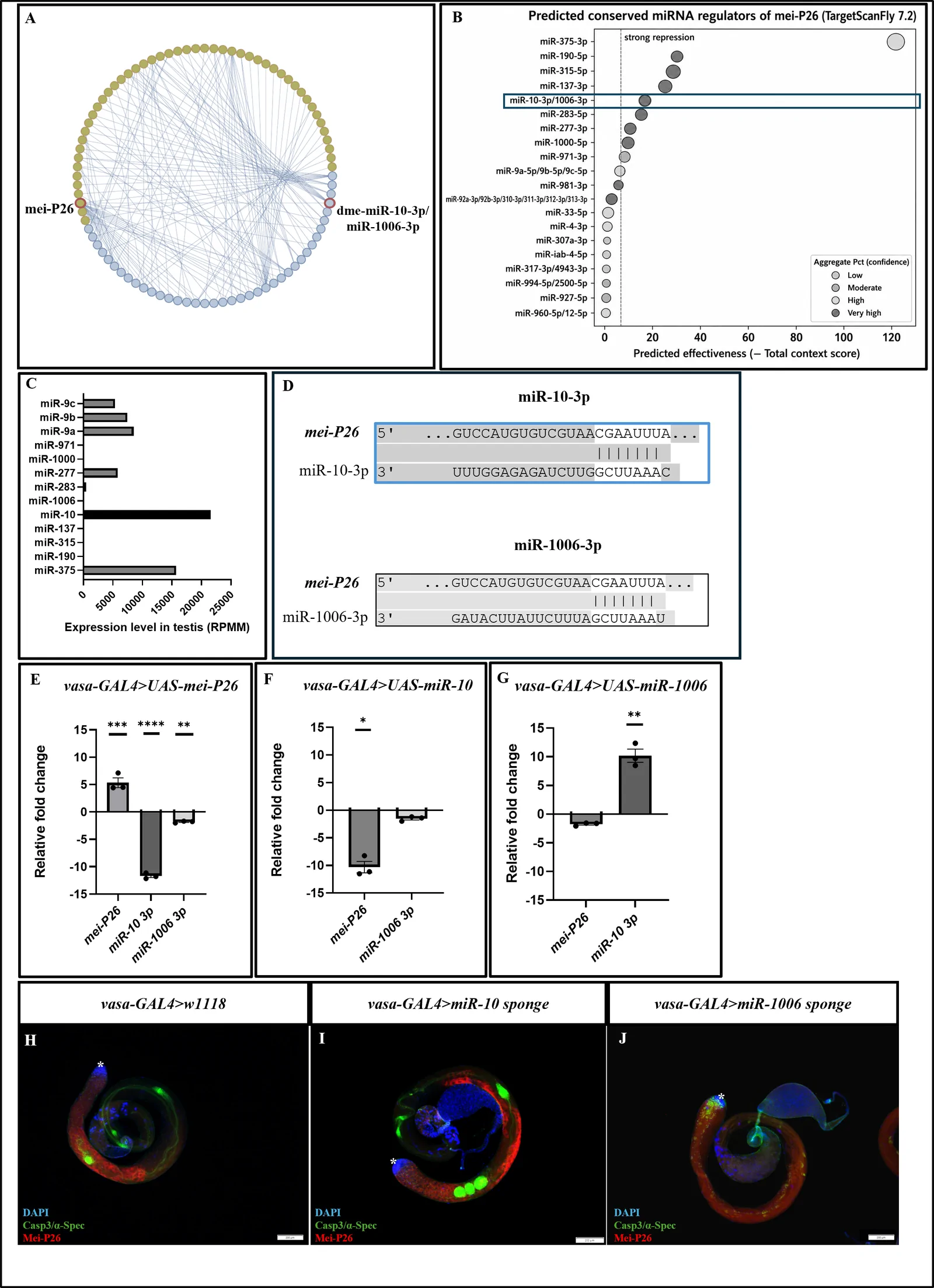



## Description

Mei-P26 is a TRIM–NHL family RNA-binding protein in *Drosophila* that functions as a post-transcriptional regulator of stem cell fate (Insco et al., 2012; Li et al., 2012; Liu et al., 2009). In the ovary, Mei-P26 acts through interactions with the miRNA pathway, including Ago1- and GW182-containing complexes, to repress target mRNA translation and maintain the balance between stem cell self-renewal and differentiation (Neumüller et al., 2008; Salerno-Kochan et al., 2022). Although this regulatory framework is well established, the contribution of individual miRNAs to Mei-P26 regulation remains poorly defined. In *Drosophila* , miRNA-mediated silencing is primarily mediated by Ago1-associated RISC complexes (Azzam et al., 2012; Förstemann et al., 2007; Lyu et al., 2014), while a subset of miRNAs is generated through Drosha-independent non-canonical pathways, producing intron-derived miRNAs termed ‘mirtrons’(Okamura et al., 2007).

Recent studies implicate Mei-P26 in neuroblast regulation during brain development (Hu et al., 2025), as well as increased Mei-P26 in proteomic analysis of adult offspring brains following paternal Western diet (Murashov et al., 2023). miRNA enrichment analysis of this proteomic dataset using MIENTURNET identified an evolutionarily conserved miRNA, miR-10-3p, as a potential regulatory candidate ( Figure 1A ) (Licursi et al., 2019; Murashov et al., 2023). Notably, miR-10-3p shares its seed sequence with the mirtron miR-1006-3p (Ruby et al., 2007), raising the possibility that these miRNAs may contribute to the post-transcriptional regulation of *mei-P26* expression ( Figure 1D ). To place these candidates in a broader regulatory context, TargetScanFly 7.2 predicted 20 conserved miRNA families with binding sites in the *mei-P26* 3′ UTR ( Figure 1B ) (Agarwal et al., 2018). Expression-based prioritization using a meta-analysis of testis RNA-seq datasets from the Eric Lai laboratory (Mohammed & Lai, 2016) revealed that miR-10 exhibits the highest testis expression among the top predicted candidates, based on reads per million mapped miRNA reads (RPMM) ( Figure 1C ). Quantitative PCR analysis showed that germline-specific overexpression of *mei-P26* resulted in a marked reduction of miR-10-3p levels, whereas miR-1006-3p levels were only modestly affected ( Figure 1E ). Reciprocally, germline-specific overexpression of miR-10 caused a significant reduction in *mei-P26* expression ( Figure 1F ), while miR-1006 overexpression produced only a minor decrease ( Figure 1G ).

Consistent with these effects, germline-specific knockdown of miR-10 led to an expansion of Mei-P26–positive spermatocytes relative to controls ( Figure 1I ) compared to control ( Figure 1H ). In contrast, germline-specific knockdown of miR-1006 did not produce a comparable increase in Mei-P26–positive spermatocytes ( Figure 1J ). Together, these results indicate that miR-10-3p, but not miR-1006-3p, plays a predominant role in regulating Mei-P26 expression in the *Drosophila* male germline. Although miR-1006-3p was expressed at substantially lower levels than miR-10-3p in the testis, it was included as a predicted *mei-P26* regulator to experimentally assess whether computationally predicted targeting is sufficient to confer functional regulation. The comparatively weaker effects observed following miR-1006 manipulation support miR-10-3p as the predominant endogenous regulator of *mei-P26* in the testis. These findings establish miR-10-3p as the principal regulator of *mei-P26* in the *Drosophila* testis. Nevertheless, the contribution of miR-1006-3p remains to be fully resolved, and future studies will be required to determine whether it plays a context-dependent or developmental stage-specific role in *mei-P26* regulation.

## Methods

Fly husbandry

All flies were raised on standard cornmeal-based food at room temperature. Crosses were raised at room temperature until eclosion and adult males with desired genotypes were shifted to 26°C for 5-7 days to ensure maximum GAL4 activity. Fly strains used in this research were obtained from Bloomington Drosophila Stock Center (BDSC) and KYOTO Stock Center (DGRC). Following fly lines were used in this study: *w* ^1118^ (BDSC# 3605), *UAS-mei-P26* (BDSC# 25771), *UAS-miR-10* (BDSC# 41169), *UAS-miR-1006* (BDSC# 41204), *UAS-miR-10-sponge* (BDSC# 61377), *UAS-miR-1006-sponge* (BDSC# 61489), and *vasa-GAL4* (DGRC# 109996).

Immunostaining

The testes of aged (5 to 7 days after eclosion) unmated F1s with desired genotype were dissected in ice cold PBSTX (1X PBS with 0.2% Triton X-100, 0.1% Tween-20). The dissected testes were fixed in 4% paraformaldehyde (PFA) for 30 minutes, washed thrice with PBSTX, and blocked in PBSTX + 5% NGS (5% normal goat serum) in PBSTX for an hour. The blocked samples were then nutated with primary antibodies at 4˚C overnight. The testes samples were then washed thrice in PBSTX, followed by blocking for one hour in PBSTX + 5% NGS before incubating with secondary antibodies at room temperature for 2.5 hours. The primary antibodies used in this study were guinea pig anti-Mei-P26 (1:400; a kind gift from Paul Lasko, McGill University) (Liu et al., 2009), mouse anti-α-Spectrin (α-Spec; 1:100; DSHB), and rabbit anti-Cleaved Caspase-3 (1:400; Cell Signaling Technology). The secondary antibodies used in this study were goat anti-rabbit IgG Alexa Fluor 488, goat anti-mouse IgG Alexa Fluor 488, and goat anti-guinea pig IgG Alexa Fluor 594 (Invitrogen). All secondary antibodies were used at dilution of 1:500. Then, the testes samples were washed thrice in PBSTX and mounted on glass slide with 10 μL SlowFade mounting medium with DAPI (Biotium).

RNA Isolation and Quantitative Real-Time PCR

Adult unmated males of 5 to 7 days in age were dissected quickly in filter sterilized ice cold PBSTX. A maximum of thirty males were processed per round. As much of the dissecting medium was removed prior to the addition of 100μl of TRIzol (Invitrogen). Tubes were immediately placed in the -80°C refrigerator for flash freezing. Three batches of testes (30 pairs each batch) were consolidated for a singular RNA extract. Total RNA from tissue samples was extracted with an RNAqueous Micro Kit (Thermo Fisher–Life Technologies) according to the manufacturer's instructions. For miRNA quantification, polyadenylation and reverse transcription were performed using Mir-X miRNA First Strand Synthesis Kit (Takara Bio; Cat. No. 638313) and for gene, first-strand cDNA was synthesized from total RNA using the SuperScript™ VILO™ cDNA Synthesis Kit (Thermo Fisher Scientific; Cat. No. 11754250). Real-time PCR reaction on cDNA was performed using the TB Green Advantage qPCR Premix Kit (Takara Bio; Cat. No. 639676) on qTOWER ^3^ iris 384 PCR System (Analytik Jena) according to the manufacturer’s instructions. For miRNAs as internal controls, primers for U6 (the noncoding small nuclear RNA) supplied with Mir-X miRNA First Strand Synthesis Kit were used and for gene, RpL32 was used as a reference gene for normalization. For specific miRNAs, miRNA-specific primer (Table 1) and mRQ 3’primer supplied with the Mir-X miRNA First Strand Synthesis Kit according to the instructions provided by the manufacturer. The relative quantifications of miRNA expression were calculated against U6 and of mRNA expression were calculated against RpL32 by the ∆∆ *C _t_^2^* method. The experiments were performed three times independently. Following are the sequence of primers used in this study:

**Table 1: List of primers used in this study.**

**Table:** 

**Primer name**	**Primer sequence (5’- 3’)**
RpL32	Forward: GACGCTTCAAGGGACAGTATCTG Reverse: AAACGCGGTTCTGCATGAG
Mei-P26	Forward: TCCGGGGATTCCCAATCTGAA Reverse: GGAGCTAGAGCTGCTAGAACT
miR-10-3p	CAAATTCGGTTCTAGAGAGGTTT
miR-1006-3p	TAAATTCGATTTCTTATTCATAG

Sequences and miRNA Target Prediction

Sequences for miR-10-3p and miR-1006-3p were recovered from miRBase.org for schematic representation. For *D. melanogaster* *mei-P26* miRNA target prediction, we used TargetScanFly 7.2 (Agarwal et al., 2018). An umbrella record for Lai lab miRNA RPMM expression values consolidated from shortRNA-seq assays of various tissues published in FlyBase release (FB2026_01) was used to plot miRNA RPMM expression in testis for top predicted *mei-P26* miRNA targets (Öztürk-Çolak et al., 2024).

## References

[R1] Agarwal V, Subtelny AO, Thiru P, Ulitsky I, Bartel DP (2018). Predicting microRNA targeting efficacy in Drosophila.. Genome Biol.

[R2] Azzam G, Smibert P, Lai EC, Liu JL (2012). Drosophila Argonaute 1 and its miRNA biogenesis partners are required for oocyte formation and germline cell division.. Dev Biol.

[R3] Förstemann K, Horwich MD, Wee L, Tomari Y, Zamore PD (2007). Drosophila microRNAs are sorted into functionally distinct argonaute complexes after production by dicer-1.. Cell.

[R4] Hu Y, Yang X, Lipshitz HD (2025). The TRIM-NHL RNA-binding protein MEI-P26 modulates the size of Drosophila Type I neuroblast lineages.. Genetics.

[R5] Insco ML, Bailey AS, Kim J, Olivares GH, Wapinski OL, Tam CH, Fuller MT (2012). A self-limiting switch based on translational control regulates the transition from proliferation to differentiation in an adult stem cell lineage.. Cell Stem Cell.

[R6] Li Y, Maines JZ, Tastan OY, McKearin DM, Buszczak M (2012). Mei-P26 regulates the maintenance of ovarian germline stem cells by promoting BMP signaling.. Development.

[R7] Licursi V, Conte F, Fiscon G, Paci P (2019). MIENTURNET: an interactive web tool for microRNA-target enrichment and network-based analysis.. BMC Bioinformatics.

[R8] Liu N, Han H, Lasko P (2009). Vasa promotes Drosophila germline stem cell differentiation by activating mei-P26 translation by directly interacting with a (U)-rich motif in its 3' UTR.. Genes Dev.

[R9] Lyu Y, Shen Y, Li H, Chen Y, Guo L, Zhao Y, Hungate E, Shi S, Wu CI, Tang T (2014). New microRNAs in Drosophila--birth, death and cycles of adaptive evolution.. PLoS Genet.

[R10] Murashov AK, Pak ES, Mar J, O'Brien K, Fisher-Wellman K, Bhat KM (2023). Paternal Western diet causes transgenerational increase in food consumption in Drosophila with parallel alterations in the offspring brain proteome and microRNAs.. FASEB J.

[R11] Neumüller RA, Betschinger J, Fischer A, Bushati N, Poernbacher I, Mechtler K, Cohen SM, Knoblich JA (2008). Mei-P26 regulates microRNAs and cell growth in the Drosophila ovarian stem cell lineage.. Nature.

[R12] Okamura K, Hagen JW, Duan H, Tyler DM, Lai EC (2007). The mirtron pathway generates microRNA-class regulatory RNAs in Drosophila.. Cell.

[R13] Öztürk-Çolak A, Marygold SJ, Antonazzo G, Attrill H, Goutte-Gattat D, Jenkins VK, Matthews BB, Millburn G, Dos Santos G, Tabone CJ, FlyBase Consortium (2024). FlyBase: updates to the Drosophila genes and genomes database.. Genetics.

[R14] Ruby JG, Jan CH, Bartel DP (2007). Intronic microRNA precursors that bypass Drosha processing.. Nature.

[R15] Salerno-Kochan A, Horn A, Ghosh P, Nithin C, Kościelniak A, Meindl A, Strauss D, Krutyhołowa R, Rossbach O, Bujnicki JM, Gaik M, Medenbach J, Glatt S (2022). Molecular insights into RNA recognition and gene regulation by the TRIM-NHL protein Mei-P26.. Life Sci Alliance.

